# Disulfiram moderately restores impaired hepatic redox status of rats subchronically exposed to cadmium

**DOI:** 10.1080/14756366.2016.1261132

**Published:** 2017-01-19

**Authors:** Aida Begic, Ana Djuric, Milica Ninkovic, Ivana Stevanovic, Dragan Djurdjevic, Milos Pavlovic, Katarina Jelic, Ana Pantelic, Goran Zebic, Bratislav Dejanovic, Ivan Stanojevic, Danilo Vojvodic, Petar Milosavljevic, Mirjana Djukic, Luciano Saso

**Affiliations:** a Department for Toxicology “Akademik Danilo Soldatović”, Faculty of Pharmacy, University of Belgrade, Belgrade, Serbia;; b Institute for Medical Research, Military Medical Academy, Belgrade, Serbia;; c Department for Reproduction, Fertility and Artificial Insemination, Faculty of Veterinarian Medicine, University of Belgrade, Belgrade, Serbia;; d Department for Pathology and Forensic Medicine, Military Medical Academy, Belgrade, Serbia;; e Department for Applied Chemistry, Faculty of Chemistry, University of Belgrade, Belgrade, Serbia;; f Department for Food Technology, Faculty of Agriculture, University of Belgrade, Belgrade, Serbia;; g Military Medical Center Karaburma, Belgrade, Serbia;; h Department of Physiology and Pharmacology, Sapienza University, Rome, Italy

**Keywords:** Cadmium, disulfiram, oxidative stress, essential metals, hepatotoxicity

## Abstract

Examination of cadmium (Cd) toxicity and disulfiram (DSF) effect on liver was focused on oxidative stress (OS), bioelements status, morphological and functional changes. Male Wistar rats were intraperitoneally treated with 1 mg CdCl_2_/kg BW/day; orally with 178.5 mg DSF/kg BW/day for 1, 3, 10 and 21 days; and co-exposed from 22nd to 42nd day. The co-exposure nearly restored previously suppressed total superoxide dismutase (SOD), catalase (CAT) and increased glutathione peroxidase (GPx) activities; increased previously reduced glutathione reductase (GR) and total glutathione-*S*-transferase (GST) activities; reduced previously increased superoxide anion radical (O_2_^·−^) and malondialdehyde (MDA) levels; increased zinc (Zn) and iron (Fe), and decreased copper (Cu) (yet above control value), while magnesium (Mg) was not affected; and decreased serum alanine aminotransferases (ALT) levels. Histopathological examination showed signs of inflammation process as previously demonstrated by exposure to Cd. Overall, we ascertained partial liver redox status improvement, compared with the formerly Cd-induced impact.

## Graphical abstract

**Figure:**
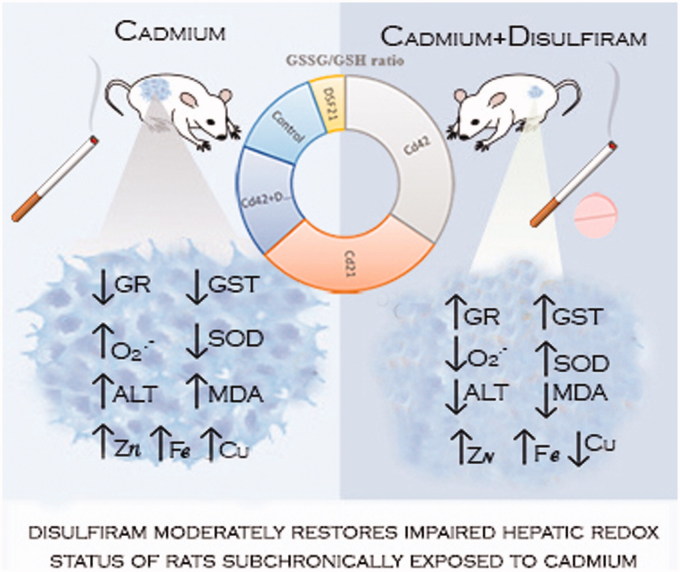

## Introduction

The raising awareness of cadmium (Cd) toxicity derives from its human carcinogenic effect (IARC Group 1) and ecotoxicological impact that is of even greater concern due to its bioaccumulation (biological half-life is up to 20 years)^1^^,^ 2. Cd is widely used in metal industry and battery manufacturing. Occupational exposure implies inhalation of dust and fumes, while public health risk is related to the contaminated food intake (aquatic organisms and leafy vegetables). Cigarette smoking increases Cd intake in humans as tobacco leaves naturally accumulate high level of Cd from the soil^3^^,^ 4. The target organ toxicity related to acute exposure to Cd involves primarily lung, liver, kidney and testes, while immunotoxicity, osteotoxicity and tumor genesis follow prolonged exposure. Cd imposes high binding affinity to sulfhydryl groups (–SH). Thus, proteins and peptides containing –SH groups have been found to be essential for its accumulation/deposition in target tissues. Therefore, metallothioneins (MTs), proteins containing –SH groups, are of great importance for Cd tissue retention, enabling its long biological half-life in the body^5^.

Reports on Cd have demonstrated its competitive antagonism with certain essential metals [copper (Cu), iron (Fe) and zinc (Zn)] for metal-binding sites in metalloenzymes. Alteration of antioxidative enzymes activities (where active metals become replaced with Cd) contributes to oxidative stress (OS) development. Overproduction of reactive species (RS) [including reactive oxygen and nitrogen species (ROS and RNS) and other free radicals (FRs)] along with insufficient antioxidative mechanisms lead to impairment of cell function and morphology including: oxidative injury of lipids, DNA and proteins. Distressed cell antioxidative capacity and energy level lead to cell signaling impairment and eventually cell death by apoptosis. Also, Cd binding to cysteines –SH group of glutathione (GSH) shifts cell redox balance towards the oxidative state^6^^,^ 7.

Disulfiram (DSF) has been used in aversive treatment of alcoholism (since 1960s) and cocaine dependence (recently). Also, Cd has been recognized as an antidote for nickel (Ni) and Cu poisoning^8^. Recent reports on DSF have demonstrated that its metabolite copper-diethyldithiocarbamate (Cu-DDTC) imposes an anticancer therapeutic effect^9^. Nucleophilic character of DDTC allows formation of stable lipophilic complex with essential metals (preferably with Cu) and also with toxic metals (such as Cd)^10–12^. Detached from antioxidant enzymes by Cd and/or chelating agent such as DDTC, transition metals such as Fe, Cu, etc. may participate in Fenton reaction, contributing to FRs overproduction and overall OS development^11^. Given that DSFs principal metabolite, DDTC, contains free –SH group, formation of Cd-DDTC was anticipated. Thus, examination of Cd interference with GSH and GSH-associated antioxidant enzymes was intriguing during the individual and/or parallel exposure to Cd and DSF.

Based on the Cd affinity for binding with –SH group containing compounds, we presumed that DSF may improve hepatic disorders accomplished by Cd, through interference with its metabolic pathways. Thus, in this study we focused on OS and essential metals-associated pathways of Cd hepatotoxicity and DSF capability to repair hepatic disturbances induced by Cd. Priority of our study was to examine if and to which extent Cd and DSF (in individual and/or parallel exposure) interfere with hepatic metal and redox status and function. Thus, we measured liver concentration of toxic Cd and essential metals such as: Cu, Fe, Zn, magnesium (Mg) and selenium (Se), and activities of the corresponding antioxidative metalloenzymes. Non-enzymatic OS parameters were also included: malondialdehyde (MDA), superoxide anion radical (O_2_^·−^), GSH, oxidized glutathione (GSSG) and GSSG/GSH ratio. Furthermore, we examined liver function and histology under the applied experimental settings.

## Materials and methods

### Animals

Experimental animals were treated according to the Guidelines for Animal Study, No. 12032014/9 (Ethics Committee of the Military Medical Academy, Belgrade, Serbia). The rats were housed in cages under standardized housing conditions (ambient temperature of 23 ± 2 °C, relative humidity of 55 ± 3% and a light/dark cycle of 13/11 h) and had free access to standard laboratory pellet food and tap water. All experiments were performed after two weeks’ period of adaptation to laboratory conditions, while the subsequent procedures were performed between 9 a.m. and 1 p.m.

### Experimental design

The experiment was conducted on male Wistar rats weighing in the range of 220–250 g. Animals were randomly divided into control group (intact group; *n* = 6), two major experimental groups: Cd- and DSF-groups, related to individual exposure for 42 and 21 days, respectively; and the experimental group co-exposed to Cd and DSF. Cd subgroups (Cd1, Cd3, Cd10, Cd21, Cd31 and Cd42; *n* = 6/subgroup) were exposed *i.p*. to 1 mg CdCl_2_/kg BW/day and DSF subgroups (DSF1, DSF3, DSF10, DSF21; *n* = 6/subgroup) were exposed orally to 178.5 mg DSF/kg BW/day for the corresponding time intervals. Rats co-exposed to Cd and DSF received *i.p.* 1 mg CdCl_2_/kg BW/day for 42 days, whereas *per os* intake of 178.5 mg DSF/kg BW/day was introduced from the 22nd day of exposure to Cd and continued for the next 21 days (Cd22 + DSF1, Cd24 + DSF3, Cd31 + DSF10, Cd42 + DSF21; *n* = 6/subgroup). The animals were anesthetized with intraperitoneal injection of 50 mg Na-pentobarbital/kg B.W. prior to being sacrificed by decapitation. Immediately removed liver was weighed and stored at −80 °C until analysis.

### Chemicals

Sodium pentobarbital Vetanarcol (0.162 g/ml) was obtained from Werfft-Chemie (Vienna, Austria). Glutathione in reduced form (98–100% purity) and oxidized form (99% purity) was obtained from Sigma-Aldrich Chemie (Steinheim, Germany), as well as orthophosphoric (OPA) and metaphosphoric acid (MPA). Sodium perchlorate monohydrate (NaClO_4_•H_2_O) was obtained from Acros Organics (Morris Plains, NJ). All sodium perchlorate solutions used as mobile phase were filtered, prior to use, through 0.45 μm cellulose filter membranes purchased from Agilent Technologies (Waldbronn, Germany). Ethylenediaminetetraacetic acid (EDTA) was obtained from Sigma-Aldrich (St. Louis, MO), whilst sodium phosphate – Na_2_HPO_4_, potassium dihydrogen phosphate – KH_2_PO_4_, trichloroacetic acid and thiobarbituric acid were obtained from Merck (Darmstadt, Germany). Nitrobluetetrazolium (NBT) and epinephrine were obtained from Sigma-Aldrich (St. Louis, MO). Ransod commercial kits were used for superoxide dismutase (SOD) determination (RANDOX Laboratories, UK). Commercial kits, used for glutathione reductase (GR) (catalog number GRSA), glutathione peroxidase (GPx) (catalog number CGP1) and glutathione-*S*-transferase (GST) (catalog number CS0410) determination, were purchased from Sigma (St. Louis, MO). Deionized water was prepared at Hospital Pharmacy Military Medical Academy (Belgrade, Serbia). Nitric (HNO_3_) and perchloric acid (HClO_4_) were obtained from Sigma-Aldrich (St. Louis, MO). Paraformaldehyde was obtained from Sigma-Aldrich, whilst hematoxylin and eosin were obtained from Merck (Kenilworth, NJ).

### Sample preparation protocol

#### Tissue preparation for glutathione determination

The liver slices were transferred into 1 ml ice-cold saline solution followed by addition of 1 ml ice-cold MPA (5% w/v) for deproteinization. Homogenization was performed twice with a Teflon pestle at 800 rpm for 15 min at 4 °C (Tehnika Zelezniki Manufacturing, Slovenia). Liver homogenates were immediately centrifuged at −4 °C and 8000 rpm for 25 min. The resulting supernatant was used for chromatographic analysis or stored at −20 °C until analysis. All tissue samples were kept on ice during the whole procedure.

#### Tissue preparation for superoxide anion radical, malondialdehyde and enzyme assays determination

The liver tissue was transferred into 1 ml of ice-cold buffered sucrose (0.25 mol/l sucrose, 0.1 mmol/l EDTA in sodium–potassium phosphate buffer, pH 7.2) and then homogenized twice with a Teflon pestle at 800 rpm for 15 min at 4 °C (Tehnika Zelezniki Manufacturing, Slovenia). Thereafter, homogenates were centrifuged at 3500 rpm for 15 min at −4 °C. Sonication was performed with supernatants (3 cycles including 30 s of sonication with a 5 s pause between cycles). The resulting supernatant was used for analysis or stored at −80 °C until analysis. All tissue samples were kept on ice during the whole procedure.

#### Tissue preparation for metal analysis

Liver tissue (close to 1 g) was mineralized with a mixture of concentrated mineral acids, nitric (HNO_3_) and perchloric acid (HClO_4_) (4:1; v/v). Following mineralization, dilution was performed with 10 ml of 0.1 M HNO_3._

#### Histopathological analysis

A portion of liver was fixed in 10% buffered formalin (pH 7.4), processed for paraffin sectioning (5 μm cross-sections) and examined by light microscopy after staining with hematoxylin (H) and eosin (E)^13^. Images were acquired using a bright-light microscope (Olympus, Tokyo, Japan) with 10–20× magnification. The morphological features of the stained sections were presented using digital images. The histological analysis was performed with the control, Cd21, Cd42, DSF21 and Cd42 + DSF21 group.

### Measurement of liver oxidative status

Parameters of oxidative status (GSH, GSSG, O_2_^·−^, MDA and activities of the following enzymes: GR, GPx, GST, SOD and CAT) were measured in liver homogenates of Wistar rats after the treatments.

#### Malondialdehyde

A terminal product of lipid peroxidation (LPO), MDA was determined spectrophotometrically by the method of Djukic et al.^6^. Following incubation of MDA with thiobarbituric acid – TBA reagent (15% trichloroacetic acid and 0.375% TBA, water solution) at 95 °C and pH 3.5, absorbance of formed red colored complex was measured at 532 nm. The results were expressed as nmol MDA/mg proteins.

#### Superoxide anion radical

Content of O_2_^·−^ was determined with a spectrophotometric method, based on the reduction of nitrobluetetrazolium – NBT to monoformazan by O_2_^·−^. The yellow color of the reduced product was measured at 550 nm^14^. The results were expressed as nmol reduced NBT/mg proteins.

#### Superoxide dismutase activity

Activity of SOD was measured using a commercial SOD kit. Spectrophotometric method is based on the reaction of superoxide radicals, generated by xanthine and xanthine oxidase (XOD), with 2-(4-iodophenyl)-3-(4-nitrophenol)-5-phenyltetrazolium chloride (I.N.T.). The absorbance of red formazan dye was measured at 505 nm. The activity of SOD is represented by a degree of inhibition and expressed as U SOD/mg proteins.

#### Catalase activity

Catalase (CAT) activity was determined according to the procedure described by Beers and Sizer^15^. Disappearance of peroxide, due to CAT catalyzed conversion of hydrogen peroxide (H_2_O_2_) into water (H_2_O) and oxygen (O_2_), was measured spectrophotometrically at 240 nm (corresponds to the enzyme activity). The results were expressed as U CAT/mg proteins. One unit of CAT decomposes one micromole of H_2_O_2_ per minute under the specified conditions.

#### HPLC-UV determination of reduced (GSH) and oxidized (GSSG) glutathione

GSH and GSSG were simultaneously measured with a high performance liquid chromatography (HPLC-UV) method. An isocratic chromatographic separation was carried out on ZORBAX Eclipse AAA (4.6 × 150 mm, 3.5 μm) analytical column (Agilent Technologies) set at a flow rate of 1 ml/min, 40 °C and 215 nm. Mobile phase consisted of 100 mM sodium perchlorate solution (pH 2.8 adjusted with 0.1% ortho-phosphoric acid). Supernatant was used for chromatographic analysis^16^. The results were expressed in regard to protein status, as nmol GSH/mg proteins or nmol GSSG/mg proteins. The GSSG/GSH ratio was also considered for the interpretation of the results.

#### Glutathione reductase activity

GR activity assay, based on the reduction of GSSG into GSH by GR, was used. GR required a donor of reducing equivalents – nicotinamide adenine dinucleotide phosphate (NADPH) for the reaction. Additionally, reduction of DTNB [5,5′-dithiobis (2-nitrobenzoic acid)] into TNB [5-thio (2-nitrobenzoic acid)] by GSH was measured spectrophotometrically at 412 nm. The increase of absorbance (corresponds to TNB formation) indicates GR activity. One unit of GR activity corresponds to the reduction of 1 μmol of DTNB into TNB. The absorbance was read with the initial delay of 60 s and every 10 s thereafter to obtain 11 time points. The samples were prepared according to the procedure specified in *Section*. ”Tissue preparation for superoxide anion radical, malondialdehyde and enzyme assays determination“. The results were expressed as U GR/mg proteins.

#### Glutathione peroxidase activity

GPx activity assay was based on the initial GSH depletion by GPx catalyzed reaction (reduction of H_2_O_2_ into H_2_O), followed by a subsequent GSSG recycling back to GSH (by GR) utilizing NADPH as a cofactor. The decrease in NADPH absorbance at 340 nm is indicative of GPx activity (since GPx is the rate limiting factor of the coupled reactions). In order to block the CAT activity (H_2_O_2_ is the substrate for both enzymes, GPx and CAT), NaN_3_ was added into the reaction mixture. The absorbance was recorded with initial 15 s delay and every 10 s thereafter to obtain 6 time points. The samples were prepared according to the procedure specified in *Section* ”Tissue preparation for superoxide anion radical, malondialdehyde and enzyme assays determination“. The results were expressed as units of U GPx/mg proteins.

#### Glutathione-S-transferase

GST activity assay utilizes conjugation of the thiol group of GSH to the 1-chloro-2-dinitrobenzene (CDNB), which results in an increase in the absorbance, proportional to the GST activity, at 340 nm. The absorbance is read immediately after preparation of the reaction tests, and every minute thereafter to obtain 6 time points. The samples were prepared according to the procedure specified in *Section* ”Tissue preparation for superoxide anion radical, malondialdehyde and enzyme assays determination“. The results were expressed as U GST/mg proteins.

### Protein determination

As results were expressed in regard to protein status, Lowry method was used for protein determination with a bovine serum albumin as a standard^17^.

### Metal content

The measurement of metals was performed with atomic absorption spectrometry with air-acetylene flame (Analyst 200, PerkinElmer). Metal standard solutions were prepared according to the PerkinElmer Pure Atomic Spectroscopy Standard Guidelines (NIST traceable CRM, AccuStandard). The absorption wavelengths were set at 228.8 nm, 216.51 nm, 213.86 nm, 305.91 nm, 285.21 nm and 196.03 nm for Cd, Cu, Zn, Fe, Mg and Se determination, respectively. Metal contents were expressed as μg/g of wet tissue (Cd, Cu, Zn, Fe, Mg) and ng/g of wet tissue (Se).

### Serum aminotransferases analysis

Serum aspartate transaminase (AST) and alanine transaminase (ALT) activities were measured by using IFCC method (without addition of pyridoxal phosphate, at 37 °C) on Advia 1200 Siemens biochemical analyzer. AST and ALT activities were expressed as U/L.

### Statistical analysis

One-way ANOVA and Tukey's *post hoc* multiple test was used (software GraphPad Prism, version 5.01) for statistical data analysis. Values are presented as the means ± standard deviation. Differences were considered statistically significant for *p* < 0.05.

## Results

Changes in liver oxidative status (MDA, O_2_^·−^, GSH, GSSG, GSSG/GSH and activities of the enzymes: GR, GPx, GST, SOD, CAT), during individual and parallel exposure of rats to Cd (for 1, 3, 10, 21, 31 and 42 days) and DSF (for 1, 3, 10 and 21 days), are presented in Tables 1, 2 and 3, respectively.

**Table 1.** Parameters of oxidative status in the liver of male Wistar rats exposed solely to Cd or DSF.

Groups	Exposure (days)	OS parameters			
		MDA (nmol/mg proteins)	O_2_^·−^ (nmol red NBT/mg proteins)	SOD (U SOD/mg proteins)	CAT (U CAT/mg proteins)
Control	0	63.6 ± 7.16	67.3 ± 6.70	2.34 ± 0.174	279.2 ± 44.10
Cd	1	101.6 ± 17.62***	116.4 ± 1.58***	2.34 ± 0.325	351.7 ± 21.79**
	3	123.8 ± 17.77***	127.9 ± 25.19***	2.18 ± 0.416	327.9 ± 47.27
	10	123.1 ± 8.95***	134.4 ± 11.39***	1.89 ± 0.264	300.0 ± 50.73
	21	134.2 ± 5.68***	139.5 ± 21.73***	1.71 ± 0.145***	209.8 ± 11.11**
	42	138.1 ± 2.45***	179.2 ± 12.04***	1.44 ± 0.286***	168.21 ± 2.17***
DSF	1	65.5 ± 11.51	131.8 ± 25.78***	1.42 ± 0.135***	196.7 ± 30.59***
	3	77.8 ± 11.44	146.1 ± 26.64***	1.39 ± 0.133***	336 ± 23.92**
	10	77.3 ± 3.31	311.3 ± 10.63***	1.19 ± 0.018***	434.7 ± 21.24***
	21	107.9 ± 10.03***	672.6 ± 14.83***	0.9 ± 0.0.072***	515.7 ± 49.45***

Label of statistical significance refers to comparison between the experimental groups and the control group (intact animals); Level of significance is labeled as follows: *(*p* < 0.01), **(*p* < 0.001), ***(*p* < 0.0001); Values are expressed as mean ± standard deviation of six separate analyses (*n* = 6).

**Table 2.** Parameters of glutathione cycle in the liver of male Wistar rats exposed solely to Cd or DSF.

Groups	Exposure(days)	OS parameters					
		GSH (nmol/mg proteins)	GSSG (nmol/mg proteins)	GSSG/GSH (ratio)	GR (U GR/mg proteins)	GST (U GST/mg proteins)	GPx (U GPx/mg proteins
Control	0	32.8 ± 3.40	1.8 ± 0.08	0.05 ± 0.004	0.0024 ± 0.0001	0.0439 ± 0.0031	1.139 ± 0.0514
Cd	1	25.6 ± 2.57**	2.1 ± 0.13*	0.08 ± 0.011	0.0019 ± 0.0002	0.0430 ± 0.0072	1.282 ± 0.1310
	3	23.5 ± 2.42***	3.7 ± 0.31***	0.16 ± 0.011	0.0019 ± 0.0001*	0.0373 ± 0.0062	1.314 ± 0.1897
	10	22.2 ± 3.99***	4.7 ± 0.38***	0.21 ± 0.050	0.0018 ± 0.0003*	0.0173 ± 0.0025***	1.620 ± 0.1783***
	21	18.3 ± 2.00***	2.1 ± 0.13*	0.11 ± 0.018	0.0017 ± 0.0002**	0.0144 ± 0.0026***	1.954 ± 0.1055***
	42	12.5 ± 2.56***	1.7 ± 0.23	0.14 ± 0.012	0.0015 ± 0.0001***	0.012 ± 0.0017***	2.067 ± 0.0777***
DSF	1	32.5 ± 5.46	4.2 ± 0.77***	0.13 ± 0.028	0.0015 ± 0.0002***	0.0450 ± 0.0057	1.228 ± 0.1469
	3	21.7 ± 3.88**	0.6 ± 0.06***	0.03 ± 0.003	0.002 ± 0.0002**	0.0578 ± 0.0037*	1.344 ± 0.1530
	10	38.0 ± 3.85	4.5 ± 0.34***	0.12 ± 0.018	0.0029 ± 0.0001*	0.0594 ± 0.0013*	1.814 ± 0.0058***
	21	31.8 ± 4.89	0.7 ± 7.10***	0.02 ± 0.007	0.0021 ± 0.0002	0.1096 ± 0.016***	2.060 ± 0.2587***

Label of statistical significance refers to comparison between the experimental groups and the control group (intact animals); Level of significance is labeled as follows: *(*p* < 0.01), **(*p* < 0.001), ***(*p* < 0.0001); Values are expressed as mean ± standard deviation of six separate analyses (*n* = 6).

**Table 3.** Parameters of oxidative status in the liver of male Wistar rats co-exposed to Cd and DSF.

OS parameters	Control	Group			
		Cd + DSF			
		0 day	1 day	3 days	10 days	21 days
MDA	63.6 ± 7.16	132.4 ± 7.10***	112.8 ± 10.51***	103.1 ± 9.98***	99.1 ± 11.64***
O_2_^·−^	67.3 ± 6.70	140.9 ± 17.39***	127.2 ± 12.4***	127.1 ± 12.49***	106.7 ± 10.97***
SOD	2.34 ± 0.174	1.51 ± 0.277***	2.13 ± 0.261	2.16 ± 0.081	2.27 ± 0.284
CAT	279.2 ± 44.10	245.4 ± 30.29	388.6 ± 57.69***	221.7 ± 12.95	216.4 ± 3.35*
GSH	32.8 ± 3.40	10.8 ± 1.94***	26.5 ± 0.42**	28.1 ± 4.24	16.3 ± 2.35***
GSSG	1.8 ± 0.08	1.9 ± 0.22	1.9 ± 0.27	1.3 ± 0.01***	1.1 ± 0.09***
GSSG/GSH	0.05 ± 0.004	0.19 ± 0.049	0.07 ± 0.01	0.05 ± 0.007	0.07 ± 0.014
GR	0.0024 ± 0.1	0.0014 ± 0.01***	0.0028 ± 0.4	0.0029 ± 0.3*	0.0026 ± 0.5
GST	0.0439 ± 0.00306	0.087 ± 0.00502***	0.087 ± 0.01097***	0.0876 ± 0.01679***	0.0893 ± 0.00115***
GPx	1.139 ± 0.0514	2.656 ± 0.001***	1.852 ± 0.0704***	1.837 ± 0.0025***	1.157 ± 0.0037

Label of statistical significance refers to comparison between the experimental groups and the control group (intact animals); Level of significance is labeled as follows: *(*p* < 0.01), **(*p* < 0.001), ***(*p* < 0.0001); Values are expressed as mean ± standard deviation of six separate analyses (*n* = 6); 21-day period refers to administration of DSF for 21 days in the combined treatment; Units of OS parameters are expressed as: MDA (nmol/mg proteins), O_2_^·−^ (nmol red NBT/mg proteins), SOD (U SOD/mg proteins), CAT (U CAT/mg proteins), GSH (nmol GSH/mg proteins), GSSG (nmol GSSG/mg proteins), GSSG/GSH ratio, GR (U GR/mg proteins), GST (U GST/mg proteins), GPx (U GPx/mg proteins).

Changes in liver metal status (Cd, Cu, Zn, Fe, Mg, Se) during individual exposure of rats to Cd (for 1, 3, 10, 21 and 42 days) and DSF (for 1, 3, 10 and 21 days), as well as during co-exposure of rats to Cd (for 22, 24, 31 and 42 days) and DSF (for 1, 3, 10 and 21 days), are presented in Table 4.

**Table 4.** Metal content in the liver of male Wistar rats.

Groups	Exposure (days)	Metal					
		Cd	Cu	Fe	Zn	Mg	Se
Control	0	/	3.3 ± 0.28	66.0 ± 12.12	37.5 ± 4.44	240.2 ± 17.35	15.2 ± 0.39
Cd	1	55.8 ± 5.48	5.6 ± 0.93**	69.4 ± 9.53	54.4 ± 8.68*	240.5 ± 22.62	41.9 ± 1.95***
	3	54.22 ± 9.43	5.3 ± 0.43**	58.5 ± 7.32	58.1 ± 5.47**	240.0 ± 11.21	32.2 ± 4.97***
	10	57.5 ± 8.68	6.3 ± 0.38***	77.3 ± 10.21	78.1 ± 6.66***	261.9 ± 48.31	28.4 ± 1.13***
	21	107.3 ± 5.93 μ***	8.4 ± 1.64***	87.0 ± 7.03***	96.7 ± 12.59***	239.7 ± 31.11	22.6 ± 2.44*
	42	166.5 ± 26.3 μ***	8.7 ± 1.19***	138.5 ± 11.95***	96.3 ± 16.94***	242.0 ± 29.96	/
DSF	1	/	3.9 ± 0.62	64.2 ± 11.65	42.5 ± 3.53	242.8 ± 19.45	18.5 ± 0.52
	3	/	5.6 ± 0.97**	57.5 ± 9.50	67.9 ± 11.44***	244.4 ± 24.41	34.9 ± 6.44***
	10	/	5.7 ± 1.18**	62.1 ± 6.87	69.3 ± 12.38***	233.8 ± 34.33	43.6 ± 7.78***
	21	/	9.0 ± 1.74***	100.1 ± 14.31***	65.6 ± 6.39**	219.7 ± 16.80	44.8 ± 8.13***
Cd + DSF	1	133.0 ± 19.46 μ***	5.7 ± 0.73**	94.7 ± 13.34***	108.6 ± 7.31***	241.6 ± 16.61	22.0 ± 0.61*
	3	106.6 ± 13.8 μ***	13.9 ± 1.61***	94.4 ± 6.23***	114.3 ± 11.23***	230.6 ± 7.72	22.3 ± 3.14*
	10	152.2 ± 29.48 μ***	9.4 ± 1.48***	102.4 ± 10.68***	145.5 ± 9.87***	241.2 ± 14.37	23.3 ± 2.03*
	21	235.3 ± 11.46 μ***	5.6 ± 0.29**	197.6 ± 11.50***	156.1 ± 14.21***	242.5 ± 31.55	24.2 ± 0.99**

Significances between experimental groups in essential metal content determination refer to comparison between the experimental groups and the control group (intact animals); Significances between experimental groups in Cd content determination refer to comparison between the experimental groups and Cd1 subgroup (μ); Level of significance is labeled as follows: *(*p* < 0.01), **(*p* < 0.001), ***(*p* < 0.0001); Values are expressed as mean ± standard deviation of six separate analyses (*n* = 6); 21-day period refers to administration of DSF for 21 days in the combined treatment; Units of metals are expressed as μg/g tissue for Cd, Cu, Fe, Zn, Mg and ng/g tissue for Se.

**Figure 1.**
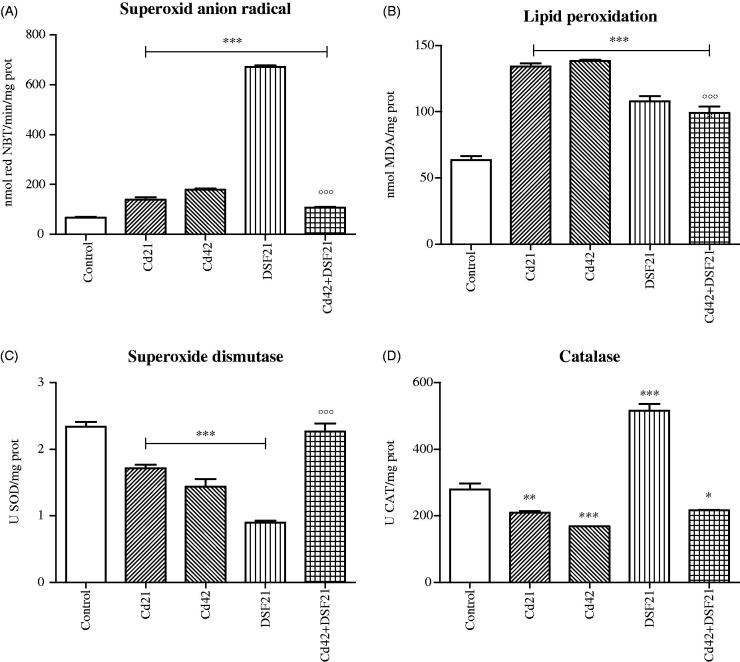
Representative graphs of oxidative status in liver of Wistar rats intact and administered Cd, DSF and a combination of both substances. *See the experimental conditions presented in the section *Experimental design*. Values are presented as means ± S.D. Labels of statistical significance: compared with the control group (*) and compared with Cd42 subgroup (°). Statistical significance was considered at: ∗*p* < 0.01, ∗∗*p* < 0.001, ∗∗∗*p* < 0.0001 and °°°*p* < 0.0001. (A) Superoxide anion radical content (O_2_^·−^): presented parameter of OS (expressed as nmol red NBT/mg proteins). (B) Lipid peroxidation: parameter of OS presented as MDA level (expressed as nmol MDA/mg proteins). (C) SOD activity: presented parameter of antioxidative defence (expressed as U SOD/mg proteins). (D) CAT activity: presented parameter of antioxidative defence (expressed as U CAT/mg proteins).

**Figure 2.**
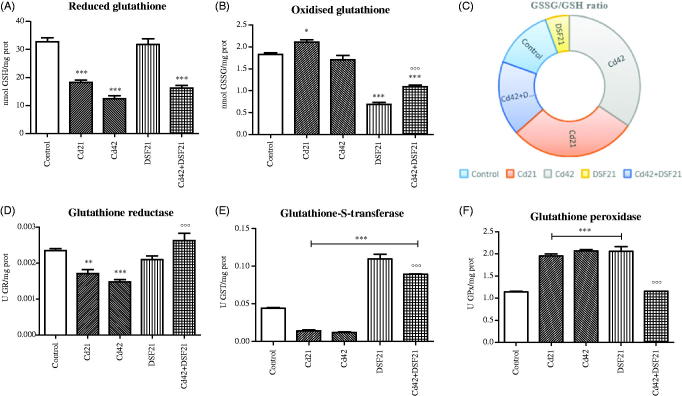
Representative graphs of glutathione cycle in liver of Wistar rats intact and administered Cd, DSF and a combination of both substances. *See the experimental conditions presented in the section *Experimental design*. Values are presented as means ± S.D. Labels of statistical significance: compared with the control group (*) and compared with Cd42 subgroup (°). Statistical significance was considered at: ∗*p* < 0.01, ∗∗*p* < 0.001, ∗∗∗*p* < 0.0001 and °°°*p* < 0.0001. (A, B) Reduced and oxidized glutathione content: presented parameters of antioxidative defence (obtained values, expressed in μM, were recalculated in regard to protein status and expressed as nmol GSH or GSSG/mg proteins for the appropriate discussion of the obtained results). (C) Ratio of oxidized and reduced glutathione: presented parameter of glutathione homeostasis (values are presented as means of GSSG/GSH ratio through chosen time points). (D) GR activity: presented parameter of antioxidative defence (expressed as U GR/mg proteins). (E) GST activity: presented parameter of antioxidative defence (expressed as U GST/mg proteins). (F) GPx activity: presented parameter of antioxidative defence (expressed as U GPx/mg proteins).

**Figure 3.**
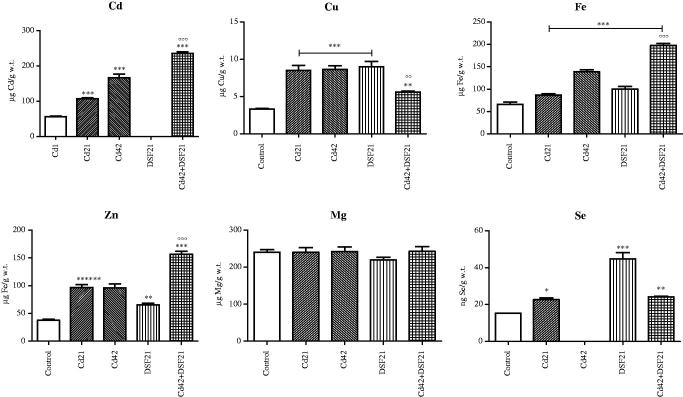
Representative graphs of metal content in liver of Wistar rats intact and administered Cd, DSF and a combination of both substances. *See the experimental conditions presented in the section *Experimental design*. Values are presented as means ± S.D. Labels of statistical significance for toxic metal Cd: compared with the Cd1 group (*) and compared with Cd42 subgroup (°). Labels of statistical significance for essential metals (Cu, Fe, Zn, Mg, Se): compared with the control group (*) and compared with Cd42 subgroup (°). Statistical significance was considered at: ∗*p* < 0.01, ∗∗*p* < 0.001, ∗∗∗*p* < 0.0001, °°*p* < 0.001 and °°°*p* < 0.0001. (A) Cd content (expressed as μg Cd/g liver tissue). (B) Cu content (expressed as μg Cu/g liver tissue). (C) Fe content (expressed as μg Fe/g liver tissue). (D) Zn content (expressed as μg Zn/g liver tissue). (E) Mg content (expressed as μg Mg/g liver tissue). (F) Se content (expressed as ng Se/g liver tissue).

Aforementioned OS-parameters and metal status for 21st and 42nd day, both for single and combined exposure to Cd and DSF are presented graphically (Figures 1–3).

**Figure 4.**
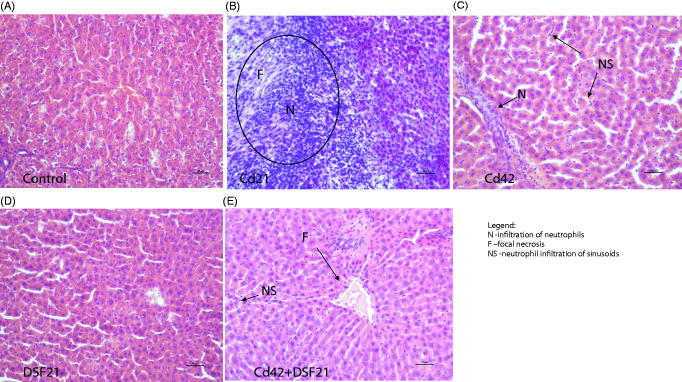
Representative photomicrographs of liver sections from Wistar rats exposed *i.p.* to 1 mg CdCl_2_/kg BW/day (for 21 and 42 days) and orally to 178.5 mg DSF/kg BW/day (for 21 days), individually and/or in combination: (A) Control/intact group, showing normal appearance; (B) Cd21: showing infiltration of neutrophils (N) and focal necrosis (F). (C) Cd42: showing infiltration of neutrophils (N) and neutrophil infiltration of sinusoids (NS). (D) DSF21: showing normal appearance; and (E) Cd42 + DSF21: group showing infiltration of neutrophils (N), neutrophil infiltration of sinusoids (NS) and focal necrosis (F).

Acquired images of stained liver sections of control and treated groups (Cd21, Cd42, DSF21 and Cd42 + DSF21) were used for histological assessment (Figure 4(A–E)).

Activity of serum aminotransferases (AST and ALT) determined at 21st and 42nd day, both for single and combined exposure to Cd and DSF, are presented in Table 5.

**Table 5.** Serum aminotransferases analysis in male Wistar rats.

Parameter	Groups				
	Control	Cd	Cd	DSF	Cd + DSF
	0 day	21 days	42 days	21 days	21 days
AST (U/L)	148 ± 15.8	184 ± 8.2¥*	230 ± 31.7¥**	136 ± 16.2	302 ± 2.5¥**;£**
ALT (U/L)	27 ± 0.6	42 ± 3.3¥**	46 ± 3.2¥**	28 ± 3.0	37 ± 2.6¥**;£**

Significances between experimental groups are labeled as follows: compared with controls (¥); compared with Cd42 subgroup (£); Level of significance is labeled as follows: *(*p* < 0.001), **(*p* < 0.0001); Values are expressed as mean ± standard deviation of six separate analyses (*n* = 6); 21-day period refers to administration of DSF for 21 days in the combined treatment.

Correlation of all measured parameters is presented in Table 6.

**Table 6.** Spearman correlation between OS parameters and/or relevant metals in the liver of the Wistar rats subacutely exposed to Cd and DSF, subchronically exposed to Cd and subchronically co-exposed to Cd and DSF.

Groups	Correlated OS parameters and metals									
Control	MDA	CAT	GSSG/GSH	Fe						
	Se	GPx	GR	Mg						
Spearman *r*	0.9429	0.9429	0.971	−0.8857						
*p* Value	0.0167	0.0167	0.0028	0.0333						
Cd21	SOD	GPx								
	GPx	MDA								
Spearman *r*	0.9276	−0.2899								
*p* Value	0.0167	0.0167								
Cd42	MDA	SAR	SOD	GST						
	Cd	GSSG	GSSG/GSH	Mg						
Spearman *r*	−0.9429	−0.9429	0.8533	−0.8827						
*p* Value	0.0167	0.0167	0.0333	0.0333						
DSF21	SAR			GSSG	GSSG/GSH	GPx				
	GSSG	GPx	Zn	Zn	GST	MDA	Zn			
Spearman *r*	−0.8697	0.9429	1	−0.8697	0.8452	−0.9429	0.9429			
*p* Value	0.0333	0.0167	0.0028	0.0333	0.0333	0.0167	0.0167			
Cd42 + DSF21	MDA		SAR	SOD		CAT	GSH		GSSG	
	SOD	GR	Zn	GSH	GR	Fe	MDA	GR	MDA	GR
Spearman *r*	−0.8857	0.9856	0.8986	0.9429	−0.9276	0.8857	−0.9429	−0.9856	0.8986	0.8676
*p* Value	0.0333	0.0028	0.0333	0.0167	0.0167	0.0333	0.0167	0.0028	0.0333	0.0333

Correlation was calculated between all parameters across all chosen time points (Cd: 21 and 42 days; DSF: 21 days and Cd 42 days and DSF 21 days of co-exposure) in Wistar rats *i.p.* receiving 1 mg CdCl_2_/kg/day and orally 178.5 mg DSF/kg BW/day. Spearman correlation coefficient (*r*) > ± 0.70 was the criteria for the segregation of the results. The units of the presented parameters are as follows: malondialdehyde (MDA): nmol MDA/mg proteins; superoxide anion radical (SAR): nmol red NBT/min/mg proteins; superoxide dismutase (SOD): units of SOD/mg proteins; catalase (CAT): units of CAT/mg proteins; glutathione – reduced and oxidized (GSH and GSSG): nmol of GSH and GSSG/mg proteins; glutathione reductase (GR): units of GR/mg proteins; Glutathione-S-transferase (GST): units of GST/mg proteins; glutathione peroxidase (GPx): units of GPx/mg proteins; and concentration of metals – iron (Fe): μg of Fe/g of wet tissue and selenium (Se): ng of Se/g of wet tissue. Differences were considered statistically significant for *p* < 0.05.

## Discussion

Guided by the fact that patients addicted to both, alcohol and nicotine, smoke more frequently due to cessation of drinking when subjected to DSF therapy, we attempted to evaluate Cd hepatotoxic effects (*via* peritoneal route) and to reveal if and to which extent DSF interferes with Cd hepatotoxic pathways, focusing on hepatic metal and redox status and function, using Wistar rats as an animal model.

Individual and parallel subacute/subchronical exposure to Cd and/or DSF resulted in Cu, Fe, Zn and Se increment in liver of the exposed rats (in all experimental groups) (Figure 3(B,C,D,F)). Additive increasing effect on hepatic Fe and Zn and moderate on Cu and Se were documented in the co-exposed group, compared with individual exposure to Cd and/or DSF.

Molecular pathway related to Cd hepatotoxicity encircles indirect OS promotion due to increase of unbound Cu and Fe ions, which participate (as transition metals, they participate in this reaction in their lower oxidation state, i.e. reduced form: Cu^+ ^and Fe^2+^) with H_2_O_2_ in Fenton reaction and contribute to hydroxyl radical (OH•) production. Cd itself is not redox active^18–20^. The other pathway includes disturbance of non-enzymatic (GSH and the total sulfhydryl groups) and enzymatic antioxidants (SOD, CAT, GPx) in the liver^7^^,^ 21 ^,^ 22. Thus, prooxidative effect of Cd was confirmed with the occurrence of antioxidative enzymes inhibition, increase of LPO and detrimental effect on GSH-cycling. DSF itself demonstrated confronting effects on hepatocellular redox buffer system compared with previous report^23^. The effect on redox system occurs as a result of nucleophilic character and metal binding affinity of its principal metabolite, DDTC, which is a thiol containing compound.

In accordance with the literature, deleterious effects of Cd during subacute/subchronic exposure on liver morphology and function were confirmed by our results^24^^,^ 25. While unable to conduct an inhalation animal study (inhalation route of Cd exposure), we administered Cd *i.p.* to male Wistar rats, since Cd delivery into the blood stream is almost equal to inhalation route^26^^,^ 27. The applied dose of 1 mg CdCl_2_/kg/day was chosen according to the literature^24^^,^ 25, while *per os* dose of 178.5 mg DSF/kg BW/day corresponds to the lowest maintaining dose of 125 mg per tablet given in therapy of recovering alcoholics^28^^,^ 29.

Initial retention of Cd in hepatocytes (Figure 3(A)) is in agreement with the literature^30^^,^ 31. The highest accumulation of Cd occurred in liver of rats co-exposed to Cd and DSF, probably as a result of DSF enterohepatic circulation and formation of Cd-DDTC, indicating that canalicular transport of Cd in hepatocytes may be linked to biliary GSH excretion^7^. This occurrence actually indicates an increased risk for liver health status in alcoholic-smokers undergoing DSF therapy. Cd accumulates in cells due to its affinity for binding to –SH groups of small peptides and proteins, such as GSH or metallothioneins (MT-I and MT-II, which expression is also incited by Cd). Confirmed time-dependent Cd liver deposition corresponds to augmentation of hepatic injury documented microscopically (Figure 4(B,C))^2^^,^ 32.

Reported Cd toxicity pathways include alteration of thiol proteins and antioxidant enzymes activity, inhibition of energy metabolism and DNA structure disruption^33^. Metalloproteins functionality critically depends on the corresponding essential metals level (Cu, Fe, Zn, Se, etc.) in close vicinity. Ion exchange between Cd and essential metals in active sites of the metalloenzymes ultimately leads to their inhibition. In fact, that particular often proposed molecular mechanism of Cd hepatotoxicity was confirmed with our results^7^. The exposure to Cd resulted in significant increase of Cu, Fe, Zn (*p* < 0.0001) and Se (*p* < 0.01) in liver (Figure 3(B,C,D,F), Table 4). As expected, gradual decrease of SOD activity (Cu is coordinately bound to four histidine residues in SOD) occurred in Cd group (Figure 1(C)). The high metal binding affinity of thiols was confirmed with Cu-DDTC formation in DSF group^34^. Moreover, increased Cu and Zn content in the liver of rats exposed individually to Cd and DSF might be explained by initiated MTs expression that also requires the release of Cu from plasma ceruloplasmine^35^. Cd has the affinity to displace Zn from MTs^36^, while DDTC causes oxidation of cysteine residues from MTs to cystine (due to capture of generated harmful oxidant radicals), which could lead to liberation of bound metal ions (Cu, Zn, Se)^37^, as seen in our results (Figure 3(B,D,F)). The interchanging between Cu/Zn and Cd, whether it takes place in SODs active site, MTs or complexes with DDTC (Cu-DDTC and Cd-DDTC), is likely the mechanism of action under the Cd and DSF co-exposure^38^. Initial increase in Se (by 175% on the first day; *p* < 0.0001) was in accordance with the reported Se redistribution from other tissues into liver upon exposure to Cd. Selenium content gradually decreased by the 21st day due to formation of Cd–Se–protein complexes (similarly to MTs) and it remained by 49% above the control value (*p* < 0.01)^39^. Significant Se increase in DSF group (*p* < 0.0001) may be explained *via* induction of MT, as it was the case with Zn^31^. Similar Se content obtained in Cd and co-exposed groups, was in both cases higher than the controls (Cd21: *p* < 0.01, Cd42 + DSF21 *p* < 0.001) (Figure 3(F), Table 4).

Time-dependent increase in Fe was achieved in all experimental groups (Cd21, Cd42: *p* < 0.0001 and DSF21: *p* < 0.0001), whereas additive effect was accomplished in co-exposed group (Figure 3(C), Table 4). Increased Fe level contributes to OS development through Fenton-like reactions^40^. Previous reports on Cd exposure alluded that Fe increase possibly occurs due to its replacement by Cd from various membrane and cytoplasmic proteins, such as ferritin and iron–sulfur clusters in Fe–S proteins^41^. Some authors reported controversial Cd effect on Fe, its decrease actually^42^. As Cd binds to sulfhydryl groups (with higher affinity than phosphate, chloride, carboxyl, or amino groups), the inactivation of key thiols affects different important biological processes i.e. causes a number of deleterious effects^7^.

Mg content was not significantly changed in any of the applied treatments (Figure 3(E), Table 4).

Antioxidative enzyme SOD converts O_2_^·−^ into H_2_O_2_ and exits in three different forms: intracellular Cu/Zn SOD (in cytosol and nucleus) and manganese (Mn) SOD (MnSOD) (in mitochondria) and an extracellular Cu/Zn-containing SOD (ecSOD), also known as SOD1, 2 and 3, respectively^7^. Total SOD (tSOD) activity represents the sum of all three isoforms. Gradual decline of tSOD activity (Cd21, Cd42, DSF21: *p* < 0.0001) (Figure 1(C), Table 1) along with instant and gradual increase of O_2_^·−^ (Cd21, Cd42, DSF21: *p* < 0.0001) (Figure 1(B), Table 1) was documented in Cd and DSF groups, which resulted in insufficient sequestration of O_2_^·−^.

Cd causes perturbation of the cytosolic and mitochondrial SOD enzyme topography by reversible binding to –SH groups of cysteine residues and by replacement of Cu and/or Mn in the active site of Cu/Zn SOD and/or MnSOD, respectively. Reduced Cu/Zn SOD activity in DSF group was a consequence of Cu-DDTC formation^43–45^. By the end of the co-exposure, tSOD activity was almost equal to the controls, despite documented markedly high O_2_^·−^ level. Assumable, the reaction between DDTC and Cd prevailed the reaction of Cu-DDTC formation, allowing SOD restauration (compared with Cd42 group, tSOD activity was significantly higher in Cd42 + DSF21 group, *p* < 0.0001) (Figure 1(C), Table 3). Increased O_2_^·−^ may be addressed to its overproduction throughout several pathways, including: (a) intramolecular radical cycling of thiols (including GSH, DDTCs, etc.), which results in an overproduction of thiyl radicals (GSS•) and O_2_^·−^ (O_2_^·−^ was in negative correlation with GSSG in Cd42 and DSF groups) (Table 6); (b) activation of Kupffer cells by Cd-mediated liver injury; (c) ALDH inhibition^46^^,^ 47; and (d) both Cd and DSF interfere with hepatic P450IIE1 enzyme, which could explain GR activity status in all experimental groups^45^^,^ 46 ^,^ 48. As Cd induces hepatic P450IIE1, it may reduce availability of nicotinamide adenine dinucleotide phosphate (NADPH) to donate reduction equivalents to GR, thus decreases GR activity^46^. On the contrary, through inhibition of hepatic P450IIE1, DSF prevents/reduces CYP2E1-mediated xenobiotic toxicity and consequent formation of O_2_^·−^, i.e. initiation of a radical chain reaction and OS development^49^.

According to the literature, an instant incline of MDA occurred in Cd groups (Cd21, Cd42: *p* < 0.0001) (Figure 1(A), Table 1)^7^. Also, significantly higher hepatic MDA levels were obtained in DSF and co-exposed group (DSF21 and Cd42 + DSF21: *p* < 0.0001; 56% higher than the controls) (Figure 1(A), Tables 1 and 3). Lipid peroxidation was significantly lower in co-exposed group than in the Cd42 group (*p* < 0.0001), alluding to the partially protective effect of DSF.

Gradual decrease of CAT with the time (Cd21: *p* < 0.001; Cd42: *p* < 0.0001; and Cd42 + DSF21: *p* < 0.01) (Figure 1(D), A.1, 3 Tables 1 and 3) occurred probably due to Cd binding to imidazole residue of His-74 in CAT^50^, implicating insufficient transformation of H_2_O_2_ into H_2_O. It is well-known that GPx converts H_2_O_2_ into H_2_O and additionally, lipid hydroperoxides (ROOH) into alcohols (ROH)^51^. Accumulation of non-processing H_2_O_2_ leaves the possibility of its homolytic cleavage into HO•, which instantly initiates LPO. Moreover, increased content of Fe and Cu in those groups supports the contribution of Fenton reaction in HO• formation (the reaction between transition metals and H_2_O_2_), supporting LPO progression (what was confirmed by our experiment)^6^. CAT activity was only elevated in DSF group (*p* < 0.0001). Averting of O_2_^·−^ sequestration by significantly reduced SOD (the lowest in DSF21 group) implies decreased H_2_O_2_ production, thus it remains unclear why CAT activity was extremely high only in this experimental group^52^.

We showed that incline of LPO was independent of GPx activity (in all experimental groups). Individual treatments with Cd and DSF resulted in gradual increase in GPx activity (Cd21, Cd42 and DSF21: *p* < 0.0001) (Figure 2(F), Table 2), opposing previous reports on Cd effect on GPx^7^. Moreover, increased GPx activity in DSF21 group may be related to the reported DDTC peroxidase-like activity (it donates electron in the reaction catalyzed by GPx)^53^. The co-exposure to DSF and Cd lead to a gradual decrease of GPx activity up to the control values, after initial increase seen on the first day, which was accordingly significantly lower compared with Cd42 group (*p* < 0.0001) (Figure 2(F), Table 3). Opposed activities of GPx (significantly high) and SOD (significantly low) were accomplished in Cd and DSF groups.

Decreased hepatic GST activity upon Cd exposure was confirmed in animal studies^7^^,^ 21 ^,^ 42. Accordingly, we obtained similar results that can be explained by Cd binding to GSH, which is the substrate for GST^6^. Gradual decline of GST activity (Cd21: *p* < 0.0001; Cd42: *p* < 0.0001) upon Cd exposure was in relation with significant GSH depletion, confirming strong binding affinity between Cd and –SH group of GSH (Cd21, Cd42 and Cd42 + DSF21 group: *p* < 0.0001) (Tables 2 and 3, Figure 2(A)). On the contrary, in the individual and parallel exposure to DSF, GSH was not affected and was accompanied with increased GST activity (DSF21, Cd42 + DSF2: *p* < 0.0001) (Figure 2(E), Tables 2 and 3), pointing out predominant Cd-DDTC formation over Cd–(GS)_2_ complex^2^.

Glutathione is a substrate for GST and donor of reducing equivalents in GPx catalyzed reaction (reduction of ROOH and H_2_O_2_, when GSH becomes oxidized into GSSG). It is also of great importance in toxic metals detoxification reactions^38^. Elevated GPx activity explains increase of GSSG and LPO in Cd groups. Additionally, significantly low GST activity in groups individually treated with Cd, indicates that GSH depletion is not on the account of thiolisation reactions (Figure 2(A,B,E,F)). It has been reported that GST catalyzes thiolisation of LPO terminal products and imposes GSH-peroxidase activity over ROOH^48^^,^ 51. Apparently, increased LPO in Cd groups may be explained by inappropriate conversion of ROOH by GST^6^. Disruption of glutathione homeostasis is actually considered to be a key element in Cd-induced hepatotoxicity^7^. Lipid peroxidation did not develop on the account of GSH depletion in DSF groups, individual or parallel treatment (Figures 1 and 2), confirming strong supportive role of DSF in glutathione cycle antioxidative defense.

Also, GSH is a prime thiol-disulphide redox buffer in cells, important for maintaining GSSG/GSH ratio balance. Flavoprotein GR catalyzes GSSG reduction back into GSH by using NADPH as a donor of electrons. Oxidized glutathione was the lowest in DSF treated groups during individual and parallel exposure (DSF21, Cd42 + DSF21: *p* < 0.0001) (Figure 2(A,B), Tables 2 and 3), indicating suppressed depletion of GSH on the account of GSSG formation. Oxidation of GSH occurs in reactions where GSH is donor of reducing equivalents (such as reaction catalyzed by GPx). These results are in line with GSSG/GSH ratio profile and decreased GR activity (Figure 2(C,D)) in DSF group. Moreover, insufficient GSSG turnover into GSH by gradually decreased GR activity occurred in Cd groups (Figure 2(A,D)) (Cd21: *p* < 0.001 and Cd42: *p* < 0.0001), which is consistent with the previous reports on Cd-induced GR inhibition^7^. Observed inconsistency in GSSG/GSH ratio within 21 days of DSF treatment might be related to changes in relation of DSF to DDTC, hence, DSF and DDTC constitute a redox couple active in biological medium (Table 2) (concomitant depletion of DDTC occurred through metal binding and thiolation reactions during daily exposure to DSF, for 21 days)^54^. Nevertheless, GSSG/GSH ratio in DSF group was significantly lower than in the controls (four times), while in Cd42 + DFS21 that value was almost double lower than in Cd42 group, indicating protective role of DSF in terms of keeping GSH reserves (Figure 2(C)). Our results are in accordance with previous reports on DDTC protection against Cd-induced hepatotoxicity afforded by DDTC chelates formation, although of low therapeutic importance because of rapid breakdown of the complex^55^.

Macroscopically, individual and prolonged exposure to Cd lead to liver enlargement and ischemia compared with the control, which is consistent with previous reports on Cd-induced liver inflammation processes that involve Kupffer cells activation and neutrophil infiltration^28^. Cd treatment also caused an increase in serum aminotransferases (AST and ALT) (Table 5), probably causing substantial leakage of hepatic enzymes into the bloodstream^21^^,^ 56. On the other hand, DSF treatment did not affect serum aminotransferases activity (Table 5), which is not in line with the previous reports on moderately elevated aminotransferases serum level during chronic DSF exposure^57^. The co-exposure to DSF and Cd resulted in decreased serum ALT activity, compared with Cd42 group (*p* < 0.0001), indicating protective role of DSF and reversible character of hepatic tissue injury induced by Cd previously.

Histopathological examination of liver from intact rats showed normal structure/microarchitecture of liver tissue (Figure 4(A)). Examination of liver from Cd21 and Cd42 groups, showed infiltration of neutrophils, which indicates the presence of inflammation (Figure 4(B,C)). Inflammation process is consistent with previous reports^22^^,^ 56 ^,^ 58. Focal necrosis is also present in Cd21 group (Figure 4(B)), which is consistent with previous report indicating appearance of hepatic necrosis at the similar applied *i.p.* dose of CdCl_2_ 59. Fat globules and signs of cytoplasmic vacuolization (nucleus shifted peripherally), which also appeared, indicate toxic damage of hepatocytes. Higher grade of neutrophil infiltration was observed in the Cd21, compared with the Cd42 group. As previously reported, repeated exposure to Cd causes acute and nonspecific chronic inflammation in parenchyma and portal tract^58^. Additionally, hepatic sinusoids enlargement and neutrophil infiltration of sinusoids were observed in Cd42 group.

Initial damage following exposure to Cd may be caused by a disturbance in thiol homeostasis and/or the production of ROS, what is in accordance with previous report indicating that Cd induces a decrease in GSH level in epithelial cells^60^. One of the possible resulting effects is an increased susceptibility to influenza viral infection, as Cd-induced OS directly increases the ability of influenza virus to replicate in the host-cell. Thus, exposure to Cd could be considered as a risk factor for individuals exposed to a greater extent to the contaminant. Further on, liver injury occurs from inflammatory processes initiated by activation of Kupffer cells, which release chemoattractants and activators of neutrophils, resulting in neutrophil influx and tissue damage. Cd interacts with vascular endothelium which leads to ischemic damage to surrounding hepatocytes. Apart from infiltration of neutrophils, hemorrhage was not shown at the applied dose of Cd^61^.

Examination of DSF21 group showed normal structure/microarchitecture of liver tissue (Figure 4(D)). Combined treatment, Cd42 + DSF21, resulted in minor infiltration of neutrophils and neutrophil infiltration of sinusoids, along with a slight sign of focal necrosis (Figure 4(E)). Combined treatment showed decrease of inflammatory infiltrate but still signs of focal necrosis compared with Cd21 group, while it remains unclear why Cd42 group showed no signs of necrosis. What is certain is that individual treatment with DSF does not disturb liver tissue,

## Conclusions

In this study, we confirmed harmful hepatic effect of Cd, in terms of disrupted redox and metals status and aminotransferases level, although possibly of reversible character at the applied *i.p*. subchronic exposure. DSFs partially protective role against Cd-associated hepatic oxidative disorders was documented by redox status improvement achieved in Cd42 + DSF21 groups, compared with Cd42 group, with an emphasis on preserving GSH reserves. Therefore, obtained results are of the great importance and may indicate DSFs beneficial effects against Cd induced OS-associated hepatic disorders in recovering alcoholic-smokers on DSF treatment.
